# Massive mesenteric panniculitis due to fibromuscular dysplasia of the inferior mesenteric artery: a case report

**DOI:** 10.1186/s12876-015-0303-5

**Published:** 2015-06-23

**Authors:** Andrew Mitchell, Véronique Caty, Yves Bendavid

**Affiliations:** 1 Departments of Anatomic Pathology and Cytology, Maisonneuve-Rosemont Hospital, 5415 Boulevard de L’Assomption, Montreal, QC H1T 2M4 Canada; 2 Department of Radiology, Maisonneuve-Rosemont Hospital, 5415 Boulevard de L’Assomption, Montreal, QC H1T 2M4 Canada; 3 Department of Surgery, Maisonneuve-Rosemont Hospital, 5415 Boulevard de L’Assomption, Montreal, QC H1T 2M4 Canada

**Keywords:** Mesentery, Panniculitis, Fibromuscular dysplasia, Visceral, Inferior mesenteric artery

## Abstract

**Background:**

Fibromuscular dysplasia (FMD) is a nonatheromatous, noninflammatory arterial disorder of unknown etiology resulting in vessel stenosis and/or aneurysm formation. The renal and cephalocervical (mainly carotid arteries) arterial beds are classically involved; involvement of visceral arteries is rare. Mesenteric panniculitis (MP) is an inflammatory process of mesenteric fat considered to be of unknown etiology. The majority of cases involve the small bowel mesentery; colorectal MP is rare. To our knowledge, no example of MP due to FMD has been described.

**Case presentation:**

A 52 year old man presented with steadily worsening lower abdominal pain. Investigation revealed ischemic rectosigmoid mucosa associated with a large mesenteric mass of unknown nature. Angiography showed the disease was limited to the distribution of the inferior mesenteric artery. Subsequent symptoms of large bowel obstruction necessitated a left hemicolectomy. Pathologic examination showed bowel wall necrosis and massive panniculitis of the rectosigmoid due to FMD. Subsequent angiographic imaging of other vascular beds was negative.

**Conclusions:**

Several features of this case are noteworthy: FMD limited to the inferior mesenteric artery has not been previously reported, FMD has not previously been implicated as a cause of MP, and the massive extent of panniculitis. An accompanying literature review of cases of visceral FMD, traditionally believed to almost exclusively affect females, highlights a greater than anticipated number of males (33 %), and a gender difference regarding concomitant involvement of cephalocervical and/or renal vascular beds (32 % in males versus 80 % in females). The latter observation may have implications regarding the value of radiologic screening of other vascular beds, particularly in asymptomatic males, in patients presenting with visceral artery FMD.

**Electronic supplementary material:**

The online version of this article (doi:10.1186/s12876-015-0303-5) contains supplementary material, which is available to authorized users.

## Background

Fibromuscular dysplasia (FMD) is a nonatheromatous, noninflammatory arterial disorder of unknown etiology resulting in vessel stenosis and/or aneurysm formation [1]. The renal and cephalocervical arterial beds are classically involved. Involvement of visceral arteries is rare. Mesenteric panniculitis (MP) is an inflammatory process of mesenteric fat, also considered to be of unknown etiology [2]. The small intestinal mesentery is by far the most common site; the colorectal mesentery is less commonly involved [2]. This case describes massive colorectal MP due to FMD of the inferior mesenteric artery (IMA), representing a unique association of two rare entities (Additional file 1).

## Case presentation

A 52-year old man came to the emergency department after ten days of steadily worsening crampy pain in the left flank and iliac fossa. His medical history was unremarkable. He had stopped smoking ten years previously (15 pack-years). The temperature was 38.8 C. Physical examination of the lower abdomen elicited some guarding, but the abdomen was otherwise supple with no palpable mass. The leucocyte count was normal. He was admitted to hospital.

An abdominal CT scan showed features compatible with colitis, probably ischemic, extending from the splenic angle to the rectum. The bowel wall was thickened with “infiltration” of the surrounding fat. A 3 cm left renal mass was also noted. Although colonoscopy showed colitis of irregular distribution, perhaps infectious, a biopsy specimen revealed normal colonic mucosa. The patient was discharged with a prescription for an antibiotic.

**Fig. 1:**
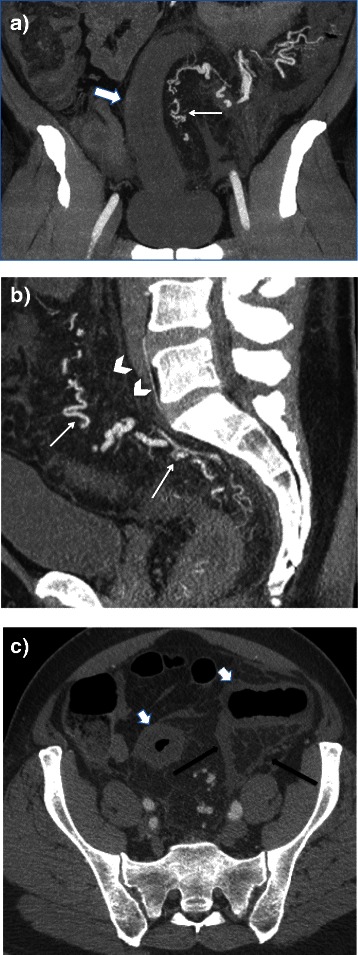
Computerized tomography images in arterial phase acquisition: **a**) Axial (1 mm slice thickness) showing fat stranding (black arrows) and thickened sigmoid wall (short thick arrows). **b** Coronal reconstruction (maximal intensity projection [MIP] slice thickness 7 mm) showing thickened recto-sigmoid wall (short thick arrow), and abnormal irregular and dilated distal vessels arising from the inferior mesenteric artery (thin arrow). **c** Sagittal reconstruction (MIP slice thickness 5 mm) showing multiple tortuous vessels (thin arrows) and hyperemic thickened mesocolon (arrow heads)

Four days later he returned to the emergency department with pain similar to that at his first admission. He was admitted to hospital. A CT scan was performed (Fig. 1) which showed a markedly thickened mesentery and a thickened, non-enhancing left colonic wall. The inferior mesenteric artery was irregular, tortuous and stenosed; there was no intravascular thrombosis. Based on these findings, vasculitis was initially considered. Colonoscopy showed marked edema of the mucosa. A biopsy specimen showed ischemic changes.

Three weeks later a diagnostic laparoscopy was performed which found ascites and multiple whitish epiploic appendices, one of which was biopsied with a subsequent microscopic diagnosis of fat necrosis. A loop colostomy was performed.

Six weeks later symptoms of large bowel obstruction developed; a left hemicolectomy with transverse colostomy was therefore performed. During the same intervention a left partial nephrectomy was carried out. The rectal stump was left open with a drain.

### Pathologic findings

The renal mass showed microscopic features characteristic of an oncocytoma.

**Fig. 2:**
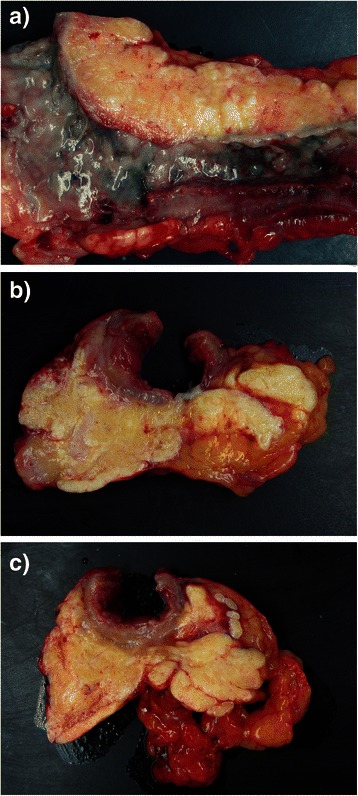
Macroscopy of the resected recto-sigmoid colon: **a**) Longitudinal view. The necrotic mucosa is covered by an extensive inflammatory pseudo-membrane. The mesentery shows diffuse fat necrosis (panniculitis). **b** and **c** Cross sectional views. The colonic wall is thickened and edematous. There is extensive mesenteric fat necrosis. Normal fat is seen at right

Macroscopic examination of the recto-sigmoid resection specimen (Fig. 2) showed diffuse hemorrhagic necrosis of the mucosa and marked bowel wall thickening with massive mesenteric necrosis. Necrotic fat encased the whole length of the resected bowel.

**Fig. 3:**
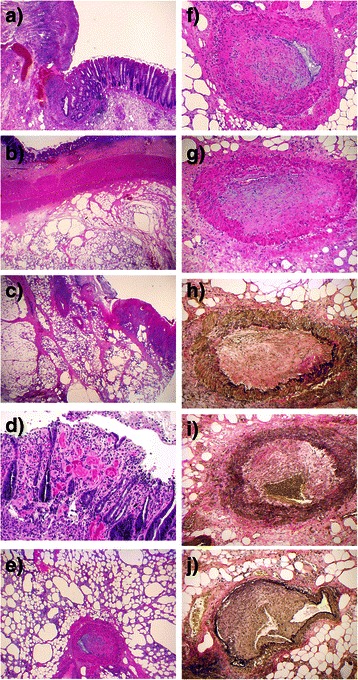
Histology of the resected recto-sigmoid colon: **a**) Scanning power view of the ulcerated mucosa with adjacent normal colonic epithelium. **b** and **c** Scanning power views of the extensively necrotic mesentery. Viable (b) and ulcerated (c) colonic mucosa are seen respectively (arrows). **d** Medium power view of colonic epithelium showing acute ischemia characterized by epithelial loss with fibrosis and multiple micro-thromboemboli within the lamina propria. **e** Medium power view of necrotic mesentery with numerous foamy macrophages. Fibrosis is absent. A medium-size vessel shows nearly complete fibrous obliteration of the lumens. **f**, **g**, **h**, **i**, **j** High power views of medium size mesenteric arteries with fibrous intimal proliferation leading to near-complete luminal occlusion: hemotoxylin-phloxin-saffrin (**f**, **g**) and Verhoff Von Geason (**h**, **i**, **j**), the latter highlighting arterial internal elastic lamina

Microscopic examination (Fig. 3) confirmed marked ischemia and ulceration of the colonic mucosa. The mesentery showed findings typical of fat necrosis. Numerous arteries and arterioles within the necrotic fat were obstructed to varying degrees, often completely, by fibrosis of the intima; the media of these vessels was normal. Atheromata, thrombosis, and inflammation were absent. The changes were considered diagnostic of fibromuscular dysplasia, intimal-type.

The postoperative course was uneventful and the patient was discharged home. Ten months later, he is asymptomatic and he is due to have his colostomy reversed.

## Conclusions

Fibromuscular dysplasia (FMD) involving visceral arteries in adults is rare (or rarely reported), with only 75 well-documented cases published since 1963 (Table 1). This is the first documented case of mesenteric panniculitis (MP) of the rectosigmoid colon due to visceral artery FMD. Two features are notable: limitation of involvement to the distribution of the inferior mesenteric artery, and the “massive” extent of the panniculitis.

**Table 1:** Reported cases of adult (greater to/equal 18 y of age) visceral artery fibromuscular dysplasia

Reference	Age/Sex	Visceral artery(s) involved^a^	Extra-visceral artery(s) involved	Histologic confirmation/artery layer
Aboumrad 1963 [8]	62, M	Celiac, SMA, IMA	Renal	Intima
Palubinskas 1964 [9]	36, F	Celiac	No	Yes: media
Ripley 1966 [10]	30, F	Celiac	No	No
Ripley 1966 [10]	50, F	SMA	Renal, common external iliac	No
Ripley 1966 [10]	45, F	SMA	Renal	No
Wylie 1966 [11]	37, F	Celiac, SMA	Renal	No
Wylie 1966 [11]	42, F	SMA	Renal	No
Wylie 1966 [11]	47, F	Celiac, SMA,	Renal	Yes: media
Wylie 1966 [11]	50, F	Celiac	Renal	No
Wylie 1966 [11]	59, F	Celiac	Renal	No
Wylie 1966 [11]	59, M	Splenic	Renal	No
Wylie 1966 [11]	68, F	Splenic	Renal	No
Wylie 1966 [11]	35, F	Celiac	Carotid	No
Wylie 1966 [11]	52, F	Celiac	No	No
Wylie 1966 [11]	73, F	Celiac, SMA	Iliac	No
Patchefsky 1967 [12]	63, F	Hepatic	Renal	Yes: intima and media
Claiborne 1970 [13]	41, F	SMA	Internal carotid, renal, iliac	Yes: media
Menanteau 1971 [14]	35, M	Jejunal	No	No
André 1973 [15]	46, M	SMA	Internal carotid,vertebral	No
André 1973 [15]	30, F	Jejunal	Renal	No
Stanley 1974 [16]	Ten patients, all F	Celiac (4), SMA (8)	Renal (eight patients)	No
Pinkerton 1976 [17]	52, M	Hepatic	No	Yes: media
Lie 1977 [18]	64, F	SMA	Circle of Willis (?)	Yes: media
Stauber 1979 [19]	65, M	Middle colic	No	Yes: intima
de Mendonca 1981 [20]	47, M	Celiac	Subclavian, renal	Yes: media
Rybka 1983 [21]	20, F	SMA, IMA	Common carotid, renal	Yes: intima
Rybka 1983 [21]	21, M	Celiac, SMA	Common carotid, internal carotid, renal	Yes: intima
Foissy 1984 [22]	55, M	SMA	No	Yes: intima
Quirke 1984 [23]	71, F	Superior rectal	No	Yes: adventitia
Kyzer 1985 [24]	33, M	Splenic	No	Yes: media
Hey 1987 [25]	38, F	Celiac	Renal, common iliac	Yes: media, adventitia
Meacham 1987 [26]	38, F	Celiac	No	No
den Butter 1988 [27]	44, F	Celiac, SMA	Aorta, renal, iliac	Yes: intima and media
Salmon 1988 [28]	58, F	Celiac, SMA, IMA	Carotid, renal	No
Insall 1992 [29]	31, F	Hepatic	Renal	No
Insall 1992 [29]	46, F	Celiac, SMA	Renal	Yes: media
Case Records…1995 [30]	60, M	SMA	No	Yes: intima and media
Stokes 1996 [31]	54, F	SMA	Coronary, renal	Yes: intima
Yamaguchi 1996 [32]	39, M	Jejunal, sigmoid	No	Yes: adventitia
Jones 1998 [33]	58, F	Hepatic	No	No
Lee 1998 [34]	23, M	Celiac, SMA	External carotid, vertebral, opthalmic, superficial temporal, renal, iliac, lumbar, intercostal	Yes: media
Safioleas 2001 [35]	33, M	SMA	No	Yes: media
Horie 2002 [36]	78, F	SMA	Coronary circumflex, renal	Yes: media and adventitia
Kojima 2002 [37]	43, M	SMA	Internal iliac	Yes: media
Felton 2003 [38]	48, F	SMA	Renal	No
Guill 2004 [39]	57, F	Celiac, SMA, IMA	No	Yes: media
Mertens 2005 [40]	48, F	Celiac, SMA, IMA	No	Yes: intima
Tsokos 2005 [41]	33, M	Splenic	No	Yes: media
Rodriguez Urrego 2007 [42]	38, M	SMA, IMA	No	Yes: intima
Chaturvedi 2008 [43]	Not provided, not provided	SMA (presumed)	No	Yes: adventitia
Kinoshita 2008 [44]	32, M	Splenic	No	Yes: intima
Malago 2007 [45]	43, F	SMA	Renal	No
Peynircioglu 2008 [46]	40, F	Common hepatic, splenic, (ileocolic ?)	Renal	No
Shussman 2008 [47]	47, F	Hepatic	Renal, iliac	No
Azghari 2009 [48]	23, M	Hepatic	No	No
Veraldi 2009 [49]	38, M	Celiac, SMA, IMA	Renal	Yes: intima and media
Watada 2009 [50]	64, M	Splenic	No	Yes: media
Kimura 2010 [51]	43, F	SMA	Renal	Yes: intima and media
Senadhi 2010 [52]	44, F	SMA	No	No
Sugiura 2011 [53]	30, M	Celiac, SMA	Renal, external iliac	Yes: media
de Gama 2012 [54]	46, M	Celiac	No	Yes:?
Dolak 2012 [55]	47, F	“All abdominal arteries”	Aorta	Yes: intima and media
Patel 2012 [56]	47, F	Celiac, SMA	No	No
Cunha 2013 [57]	27, M	Celiac	No	Yes: ?
Sekar 2013 [58]	19, F	SMA	Renal	Yes: not specified
Ünlü 2014 [59]	60, F	Celiac	Renal, external iliac	Yes: media
Present case	52, M	IMA	No	Yes: intima

^a^Smaller arteries are specified only in the absence of Celiac, SMA and IMA trunk involvement

*SMA* superior mesenteric artery, *IMA* inferior mesenteric artery

Mesenteric panniculitis (MP) is a rare inflammatory disorder leading to “tumorlike” enlargement of the mesentery, the vast majority of cases involving the small bowel [2, 3]. It is characterized by variable degrees of fat necrosis, chronic inflammation and fibrosis. This histologic variability has resulted in other terms which reflect the dominant morphologic finding, including sclerosing mesenteritis and mesenteric lipodystrophy [2]. Numerous clinical associations have been noted and there are many theories regarding etiology [3]. No unifying pathophysiologic mechanism has been elucidated, likely because it is the result of a number of disease processes.

FMD is defined as a “nonatheroscleotic, noninflammatory vascular disease that may result in arterial stenosis, occlusion, aneurysm, or dissection [1]”. The renal and cervicocranial (CC) vascular beds are classically involved [1]. A landmark consensus histologic classification of renal artery FMD was published in 1971, emphasizing the vessel layer involved [4]: 1) intimal fibroplasia (1-2 % of cases), 2) medial, of which there are four subtypes: medial dysplasia with mural aneurysm (60-70 %), medial hyperplasia (5-15 %), perimedial fibroplasia (15-25 %), medial dissection (5-10 %), and 3) periarterial fibroplasia (less than 1 %). Subsequent experience has shown that intimal FMD is more common than was appreciated in 1971, and that there are reliable angiographic correlates to these histologic subtypes [1]. It is noteworthy that the opportunity for microscopic examination of involved vessels in suspected FMD is now exceptional: in a recent review of 447 patients, tissue for pathologic analysis was available in only 3.3 % of cases (from all vascular beds). The diagnosis is now established in essentially all cases by angiographic and noninvasive imaging [1].

The angiographic appearance of medial FMD is classically described as “string-of-beads” (typical FMD) [5] and is secondary to medial thickening causing luminal stenosis, alternating with zones of mural thinning and dilatation (aneurysms), the latter associated with loss of the internal elastic lamina [1, 4, 5]. In certain vascular beds, and, of note, the mesenteric vessels, the string-of-beads change is less frequent. Rather, the angiographic appearance is one of tubular constriction (atypical FMD) [1, 5], which correlates with intimal involvement, such as was present here. Thus, absence of the string-of-beads sign should not reflexively rule out a diagnosis of FMD.

A 2014 consensus document [6] recommends, when either renal or cervicocranial FMD is discovered, screening of the “other” vascular bed provided identification of any new lesion will modify the patient’s management. The authors extend this recommendation to screening of “less often involved vascular beds” only when there are suggestive symptoms or a suggestive medical history. Angiographic imaging had revealed no involvement of the renal arteries in our patient. In light of the diagnosis of FMD of a major visceral artery, angiographic study of the CC vessels was subsequently performed which revealed no abnormality. In retrospect, this might have been expected as our summary of the reported cases of FMD of visceral arterial beds (Table 2) shows that only 12 % of males with visceral FMD had CC involvement.

**Table 2:** Summary of reported cases of visceral fibromuscular dysplasia

	Male	Female
Number of cases^a^	25	50
Artery		
Celiac	3	9
SMA	5	12
IMA	1	-
Any combination of Celiac, SMA, IMA	6	21
Other^b^		
Hepatic	2	4
Splenic	5	1
Hepatic and splenic	-	1
Jejunal	1	1
Jejeunal and sigmoid	1	-
Middle colic	1	-
Superior rectal	-	1
Cephalocervical/renal involvement		
None	17	10
Cephalocervical	1	2
Renal	5	36
Both	2	2
Vessel layer		
Intima	7	3
Media	9	7
Intima and media	2	4
Media and adventitia	-	2
Adventitia	1	1
Not specified/not stated	6	33

^a^Chaturvedi (2008) [43], and Cormier (2005) [7]

^b^Smaller arteries are specified only in the absence of Celiac, SMA and IMA trunk involvement

*SMA* superior mesenteric artery, *IMA* inferior mesenteric artery

Table 2 also highlights that, although the earliest reports show an overwhelming majority of cases occurring in females, later cases document many more males with visceral FMD, such that men now represent one third of cases. There is also a gender difference regarding associated CC and renal artery involvement: concomitant CC and/or renal artery disease was present in 80 % of females, whereas these vessels were involved individually or together in 32 % of men. Involvement of the IMA is highly unusual; indeed, we describe the first case in which disease is limited to this artery.

Of note, regarding the reported cases of visceral FMD, we must mention an extraordinary publication from 2005 [7] which tallies 30 cases of FMD of the SMA from one Parisian clinic alone, which would thus account for more than one third of all the reported cases retrieved through the PubMed database. As this uniquely vast experience does not sufficiently detail individual patients and includes presentations not described elsewhere (e.g. SMA dissection in eight patients), we have chosen to exclude it from the table.

In conclusion, we describe FMD as the etiology of a case of massive colorectal MP. FMD of visceral arteries may have “atypical” clinical and radiologic features, and, although a rare entity, should be considered when MP is diagnosed.

## Consent

Written informed consent was obtained from the patient for publication of this case report and any accompanying images. A copy of the written consent is available for review by the Editor-in-Chief of this journal.

## Additional file

Additional file 1:
**CARE Checklist (2013) of information to include when writing a case report.**

## Abbreviations

CC: Cephalocervical
FMD: Fibromuscular dysplasia
MP: Mesenteric panniculitis
SMA: Superior mesenteric artery
IMA: Inferior mesenteric artery

## References

[1] Olin JW, Froehlich J, Gu X, Bacharach JM, Eagle K, Gray BH, Jaff MR, Kim ES, Mace P, Matsumoto AH, McBane RD, Kline-Rogers E, White CJ, Gornik HL (2012). The United States Registry for fibromuscular dysplasia. Results in the first 447 patients. Circulation.

[2] Emory TS, Monihan JM, Carr NJ, Sobin LH (1997). Sclerosing mesenteritis, mesenteric panniculitis and mesenteric lipodystrophy: a single entity?. Am J Surg Path.

[3] Akram S, Pardi DS, Schaffner JA, Smyrk TC (2007). Sclerosing mesenteritis: clinical features, treatment, and outcome in ninety-two patients. Clin Gastroenterol Hepatol.

[4] Harrison EG, McCormack LJ (1971). Pathologic classification of renal artery disease in renovascular hypertension. Mayo Clin Proc.

[5] Lüscher TF, Lie JT, Stanson AW, Houser OW, Hollier LH, Sheps SG (1987). Arterial fibromuscular dysplasia. Mayo Clin Proc.

[6] Persu A, Giavarini A, Touzé E, Januszewicz A, Sapoval M, Azizi M, Barral X, Jeunemaitre X, Morganti A, Plouin PF, de Leeuw P (2014). European consensuson the diagnosis and management of fibromuscular dysplasia. J Hypertens.

[7] Cormier F, Cormier JF (2005). Trente-huit cas de lesions dysplasiques de l'artere mesenterique superieure. J Mal Vasc.

[8] Aboumrad MH, Fine G, Horn RJ (1963). Intimal hyperplasia of small mesenteric arteries. Arch Pathol.

[9] Palubinskas AJ, Ripley HR (1964). Fibromuscular hyperplasia in extrarenal arteries. Radiology.

[10] Ripley HR, Levin SM (1966). Abdominal angina associated with fibromuscular hyperplasia of the celiac and superior mesenteric arteries. Angiology.

[11] Wylie EJ, Binkley FM, Palubinskas AJ (1966). Extrarenal fibromuscular hyperplasia. Am J Surg.

[12] Patchefsky AS, Paplanus SH (1967). Fibromuscular hyperplasia and dissecting aneurysm of the heparic artery. Arch Path.

[13] Claiborne TS (1970). Fibromuscular hyperplasia; report of a case with involvement of multiple arteries. Amer J Med.

[14] Menanteau B, Segal S, Segal M (1971). Hyperplasie fibro-musculaire des artères jéjunales. Ann Med Reims.

[15] André JM (1973). Dysplasies fibreuses des artères. Aspects cliniques. Les dysplasies casculaires systématiques.

[16] Stanley JC, Gewertz BL, Bove EL, Sottiurai V, Fry WJ (1975). Arterial fibrodysplasia: histopathologic character and current etiologic concepts. Arch Surg.

[17] Pinkerton JA, Wood WG, Fowler D (1976). Fibrodysplasia with dissecting aneurysm of the hepatic artery. Surgery.

[18] Lie JT, Kim HS (1977). Fibromuscular dysplasia of the superior mesenteric artery and coexisting cerebral berry aneurysms. Angiology.

[19] Stauber R, Gerstner L (1979). Rupturiertes aneuerysma der a. colica media. Chirurg.

[20] de Mendonca WC, Espat PA (1981). Pheochromocytoma associated with arterial fibromuscular dysplasia. Am J Clin Pathol.

[21] Rybka SJ, Novick AC (1983). Concomitant carotid, mesenteric and renal artery stenosis due to primary intimal fibroplasia. J Urol.

[22] Foissy P, Fabre M, Lebaleur A, Buffet C, Frileux C, Étienne JP (1984). Anévrysme du tronc de l'artere mésentérique supérieure et maladie polyanévrysmale de l'arcade bordante paracolique droite à type d'hyperplasie fibro-musculaire. Ann Med Interne.

[23] Quirke P, Campbell I, Talbot IC (1984). Ischaemic proctitis and adventitial fibromuscular dysplasia of the superior rectal artery. Br J Surg.

[24] Kyzer S, Bayer I, Turani H, Lewinsky U, Chaimoff C (1985). Spontaneous rupture of the splenic artery as a presenting symptom of fibromuscular dysplasia of the artery. Acta Chir Iug.

[25] Hey A, Röckelein G (1987). Arterial fibromuscular dysplasia as an unexpected cause of death in adults. Dtsch Med Wochenschr.

[26] Meacham PW, Brantley B (1987). Familial fibromuscular dysplasia of the mesenteric arteries. South Med J.

[27] den Butter G, van Bockel JH, Aarts JCNM (1988). Arterial fibrodysplasia: rapid progression complicated by rupture of a visceral aneurysm into the gastrointestinal tract. J Vasc Surg.

[28] Salmon PJM, Allan JS (1988). An unusual case of fibromuscular dysplasia. J Cardiovasc Surg.

[29] Insall RL, Chamberlain J, Loose HWC (1992). Fibromuscular dysplasia of visceral arteries. Eur J Vasc Surg.

[30] Case records of the Massachusetts General Hospital (1995). Weekly clinicopathological exercises. Case 9-1995. A 60-year-old man with hypertrophic cardiomyopathy and ischemic colitis. N Engl J Med.

[31] Stokes JB, Bonsib SM, McBride JW (1996). Diffuse intimal fibromuscular dysplasia with multiorgan failure. Arch Intern Med.

[32] Yamaguchi R, Yamaguchi A, Isogai M, Hori A, Kin Y (1996). Fibromuscular dysplasia of the visceral arteries. Am J Gastroenterol.

[33] Jones HJ, Staud R, Williams RC (1998). Rupture of a hepatic artery aneurysm and renal infarction: 2 complications of fibromuscular dysplasia that mimic vasculitis. J Rheumatol.

[34] Lee EK, Hecht ST, Lie JT (1998). Multiple intracranial and systemic aneurysms associated with infantile-onset arterial fibromuscular dysplasia. Neurology.

[35] Safioleas M, Kakisis J, Manti C (2001). Coexistance of hypertrophic cardiomyopathy and fibromuscular dysplasia of the superior mesenteric artery. N Engl J Med.

[36] Horie TH, Seino Y, Miyauchi Y, Saitoh T, Takano T, Ohashi A, Yamada N, Tamura K, Yamanaka N (2002). Unusual petal-like fibromuscular dysplasia as a cause of acute abdomen and circulatory shock. Jpn Heart J.

[37] Kojima A, Shindo S, Kubota K, Iyori K, Ishimoto T, Kobayashi M, Tada Y (2002). Successful surgical management of a patient with multiple visceral artery aneurysms due to fibromuscular dysplasia. Cardiovasc Surg.

[38] Felton TW, Drewe E, Jivan S, Hall RI, Powell RJ (2003). A rare case of shock. Ann Rheum Dis.

[39] Guill CK, Benavides DC, Rees C, Fenves AZ, Burton EC (2004). Fatal mesenteric fibromuscular dysplasia. Arch Intern Med.

[40] Mertens J, Daenens K, Fourneau I, Marakbi A, Nevelsteen A (2005). Fibromuscular dysplasia of the superior mesenteric artery-case report and review of the literature. Acta Chir Belg.

[41] Tsokas M, Nolting RO, Lockemann U (2005). Sudden, unexpected death due to splenic artery aneurysm rupture. Am J Forensic Med Pathol.

[42] Rodriguez Urrego PA, Flanagan M, Tsai WS, Rezac C, Barnard N (2007). Massive gastrointestinal bleeding: an unusual case of asymptomatic extrarenal, visceral, fibromuscular dysplasia. World J Gastroenterol.

[43] Chaturvedi R, Vaideeswar P, Joshi A, Pandit S (2008). Unusual mesenteric fibromuscular dysplasia-a rare cause for chronic intestinal ishaemia. J Clin Pathol.

[44] Kinoshita H, Kubota A, Kasuda S, Nishiguchi M, Takahashi M, Ouchi H, Minami T, Otsu N, Yoshida S, Adachi N, Matsui K, Yamamura T, Motomura H, Hishida S (2008). An autopsy case of rupture of an aneurysm of the splenic artery. Soud Lek.

[45] Malago R, D'Onofrio M, Mucelli RP (2008). Fibromuscular dysplasia: noninvasive evaluation of unusual case of renal and mesenteric involvement. Urology.

[46] Peynircioglu B, Cil BE (2008). Standing waves of hepatic artery associated with renal and extrarenal fibromuscular dysplasia. Cardiovasc Intervent Radiol.

[47] Shussman N, Edden Y, Mintz Y, Verstandig A, Rivkind AI (2008). Hemobilia due to hepatic artery aneurysm as the presenting sign of fibro-muscular dysplasia. World J Gastroenterol.

[48] Azghari A, Elouannani M, Echarrab M, Elalami F, Amraoui M, Errougani A, Chkof MR (2009). Hemobilia due to fibromuscular dysplasia of the hepatic artery. Gastroenterol Clin Biol.

[49] Veraldi GF, Zecchinelli MP, Furlan F, Genco B, Minicozzi AM, Segattini C, Pacca R (2009). Mesenteric revascularisation in a young patient with antiphospholipid syndrome and fibromuscular dysplasia. Chir Ital.

[50] Watada S, Obara H, Shimoda M, Matsubara K, Matsumoto K, Kitajima M (2009). Multiple aneurysms of the splenic artery caused by fibromuscular dysplasia. Ann Vasc Surg.

[51] Kimura K, Ohtake H, Kato H, Yashiki N, Tomita S, Watanabe G (2010). Multivisceral fibromuscular dysplasia: an unusual case of renal and superior mesenteric involvement. Ann Vasc Dis.

[52] Senadhi V (2010). A rare case of chronic mesenteric ischemia from fibromuscular dysplasia: a case report. J Med Case Rep.

[53] Sugiura T, Imoto K, Uchida K, Yanagi H, Machida D, Okiyama M, Yasuda S, Takebayashi S (2011). Fibromuscular dysplasia associated with simultaneous spontaneous dissection of four peripheral arteries in a 30-year-old man. Ann Vasc Surg.

[54] da Gama AD, Ministro A, Cabral G, Pestana C, Oliveira P (2012). Surgical management of a spontaneous dissection of the celiac axis caused by fibromuscular dysplasia. First clinical report. Rev Port Cardiotorac Vasc.

[55] Dolak W, Maresch J, Kainberger F, Wrba F, Müller C (2012). Fibromuscular dysplasia mimicking Crohn’s disease over a period of 23 years. J Crohns Colitis.

[56] Patel NC, Palmer WC, Gill KRS, Wallace MB (2012). A case of mesenteric ischemia secondary to fibromuscular dysplasia (FMD) with a positive outcome after intervention. J Interv Gastroenterol.

[57] Cunha E, Sà D, Rosa A, Carmo GD, Costa T, Pestana C, Horta A, Catarino A, Da Gama AD (2013). Abdominal angina due to fibromuscular dysplasia of the celiac axis. Surgical management. Rev Port Cardiotorac Vasc.

[58] Sekar N, Shankar R (2013). Fibromuscular dysplasia with multiple visceral artery involvement. J Vasc Surg.

[59] Unlü C, van den Heuvel DA, Leeuwis JW, de Vries JP (1799). Ruptured aneurysm of the splenic artery associated with fibromuscular dysplasia. Ann Vasc Surg.

